# Hemorrhage from varices in hepaticojejunostomy in the fifth and tenth year after surgery for hepatic hilar bile duct cancer: a case report

**DOI:** 10.1186/1757-1626-1-59

**Published:** 2008-07-25

**Authors:** Hiroki Taniguchi, Michihisa Moriguchi, Hisashi Amaike, Nobuaki Fuji, Yasutoshi Murayama, Toshiyuki Kosuga

**Affiliations:** 1 Division of Digestive Surgery, Department of Surgery, Kyoto Prefectural University of Medicine, Kawaramachi-Hirokohji, Kamigyo-ku, Kyoto 602-8566, Japan; 2 Department of Diagnostic Radiology, Shizuoka Cancer Center Hospital, Nagaizumi, Shizuoka 411-8777, Japan; 3 Department of Surgery, Kameoka Municipal Hospital, Shino-Noda 1-1, Kameoka City, Kyoto 621-8585, Japan; 4 Department of Surgery, Kyoto Prefectural Yosanoumi Hospital, Otokoyama, Yosano-cho, Kyoto 629-2261, Japan

## Abstract

We report a case of a 64-year-old female patient who underwent a right lobectomy of the liver (including total resection of the caudate lobe), dissection of the group 2 lymph nodes, left hepaticojejunostomy (Roux-en-Y fashion), and reconstruction of the portal vein (end-to-end anastomosis between the main portal vein and the left portal branch) for treatment of hepatic hilar bile duct cancer in 1996. In 2001, the anastomotic site of the hepaticojejunostomy was dissected and re-anastomosed due to gastrointestinal bleeding caused by variceal rupture in the jejunal loop. In 2006, splenectomy was performed for recurrence of gastrointestinal bleeding due to another variceal rupture in the jejunal loop. Portal venography performed perioperatively showed a decrease in portal blood flow into the liver via the jejunal varices and an increase in portal blood flow into the liver via the left gastric vein. She had two jejunal variceal ruptures at five-year intervals after extrahepatic portal obstruction and underwent successful treatments.

## Introduction

Bleeding from varices in the jejunal loop due to extrahepatic portal obstruction after reconstruction of the biliary tract is rare, and the treatment of such bleeding is difficult and the prognosis is poor. Here, we report a case in which a patient is alive and well 11 years after surgery for bile duct cancer in the hepatic hilus after undergoing two operations for postoperative variceal rupture in the afferent jejunal loop.

## Case presentation

The patient was a 64-year-old female who underwent a right lobectomy of the liver (including total resection of the caudate lobe), dissection of the group 2 lymph nodes, left hepaticojejunostomy (Roux-en-Y fashion), and reconstruction of the portal vein (end-to-end anastomosis between the main portal vein and the left portal branch) for treatment of bile duct cancer in the hepatic hilus on July 25, 1996. Operative bleeding was 1,830 g and the operation time was 275 minutes. Autologous blood transfusion was performed with 400 g of blood collected from the patient preoperatively, but no homologous blood transfusion was used. There were no specific postoperative problems such as anastomotic leakage, and the patient was discharged from hospital on the 54th day of hospitalization. Postoperative biochemical laboratory findings are shown in Table 1.

**Table 1:** Laboratory data before and after the first operation

	**POD 0**	**POD 1**	**POD 3**	**POD 7**
**LDH (IU/l)**	198	281	188	135
**GOT (IU/l)**	27	144	114	15
**GPT (IU/l)**	23	68	131	33
**Total bilirubin (mg/dl)**	0.58	0.78	1.64	0.94

POD: post operative day

Since abnormal neovascularization of the portal vein in the hepatic hilus was confirmed in CT 1 year after surgery, portal venography via the superior mesenteric artery was performed and obstruction of the portal vein was observed. Percutaneous transhepatic portography was performed, but it was impossible to insert an expandable stent. Subsequently there were no problems or recurrence, but the patient began to complain of melena in early July 2001. Upper gastrointestinal endoscopy showed no bleeding points such as esophageal/gastric varices and ulcerous lesions, but small-bowel endoscopy showed clots in the anal side of the jejunum where jejunojejunostomy (Roux-en-Y) had been performed. Thus, an emergency operation was performed on July 10, 2001, on suspicion of hemorrhage from the jejunal varices developed close to the site of the hepaticojejunostomy. The blood hemoglobin and hematocrit contents on July 9 were 7.9 g/dl and 23.8%, respectively. Intraoperative endoscopy on the jejunal stump of the hepaticojejunostomy confirmed the presence of bleeding varices with a red colour sign close to the jejunal side at the site of the hepaticojejunostomy. Since the hemorrhage level increased rapidly during surgery and became uncontrollable, dissection of the hepaticojejunostomy was performed as preparation for postoperative hepatic failure. After hemostasis was achieved, an endoscopic examination of the intrahepatic bile duct showed no recurrence of cancer or varices. Hepaticojejunostomy was performed again and the surgery was completed. Postoperatively, bilirubin adsorption was performed 6 times. Total bilirubin reached its highest value (13.6 mg/dl) on July 16 (POD 6), but had decreased by August 1 (POD 22) and returned to the preoperative level in September. The patient was discharged from hospital on September 8 (POD 60). A hepaplastin test showed the lowest value (36%) on July 18.

**Figure 1:**
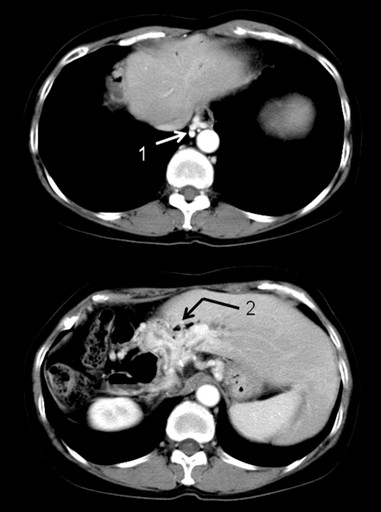
**X-ray computed tomography before the third operation.** Esophageal varices (1) were noted (upper panel) and neovascularization of the portal vein (2) is visible in the hepatic hilar area.

The patient had no major problems until melena developed again on November 18, 2006, and thus a close examination was performed. Upper gastrointestinal endoscopy showed no esophageal/gastric varices and no ulcerous lesions. Lower gastrointestinal endoscopy showed clots and dilated capillary vessels in the colon, but no varices and ulcerous lesions. Abnormal growth of the portal vein in the hepatic hilus was confirmed on CT (Figure 1), and although esophageal varices were apparent, they did not appear on the mucous surface. Bleeding points could not be confirmed by red blood cell scintigraphy, but angiography via the superior mesenteric artery showed a significantly dilated jejunal vein and communication with the intrahepatic portal vein. The left gastric vein was visible following the superior mesenteric vein, with confirmation of a collateral route that was thought to flow from the side of the lesser curvature of the stomach to the azygos vein via esophageal varices. This suggested communication between the left gastric vein and the intrahepatic portal vein. Angiography via the celiac trunk detected the left gastric vein following the splenic vein, with a collateral circulation route that was also thought to flow from the side of the lesser curvature of the stomach to the azygos vein via esophageal varices. Based on these findings, melena was diagnosed as hemorrhage from jejunal varices that had developed upstream of a newly developed jejunal vein/intrahepatic portal anastomotic branch. Blood hemoglobin and hematocrit decreased to 5.8 g/dl and 18.4%, respectively, on December 11, 2006, but these values improved after homologous blood infusion.

**Figure 2:**
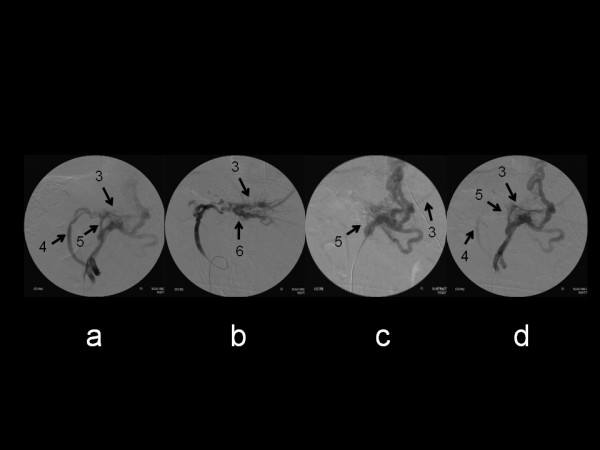
**Intraoperative venography via the ileocecal vein.** a:Detection of intrahepatic portal veins (3) supplied from the jejunal vein (4) and the left gastric vein (5). b: Intraoperative venography via the jejunal vein. Intrahepatic portal veins (3) communicated with the jejunal vein via varicose veins (6). c: Intraoperative venography via the left gastric vein. Intrahepatic portal veins (3) communicated with the left gastric vein (5), but the blood flow was thought to be small. d: Intraoperative venography via the ileocecal vein just before the operation was complete. Detection of intrahepatic portal veins (3) supplied wholly from the left gastric vein (5). The blood flow via jejunal varices (4) decreased in volume.

Splenectomy and intraoperative portography were performed on January 18, 2007. After the splenectomy, portal venography performed by inserting a catheter from the ileocolic vein showed communication from both the dilated jejunal vein and the left gastric vein to the intrahepatic portal branch (Figure 2a), and venography performed from the jejunal vein showed communication between the intrahepatic portal vein and the moniliform jejunal vein (Figure 2b). Angiography performed from the superior mesenteric vein (Figure 2c) and the ileocolic vein (Figure 2d) confirmed that the surgery was complete, since the imaging intensity of the dilated jejunal vein decreased and that of the intrahepatic portal vein from the left gastric vein increased. The postoperative prognosis was good and the patient was discharged from hospital on January 30 (POD 12).

## Discussion

The prognosis of jejunal varices is generally poor[1]. Hemorrhage from varices in the jejunal loop due to extrahepatic portal obstruction after reconstruction of the biliary tract by a procedure such as pancreatoduodenectomy has been reported[2,3]. Treatment of this kind of hemorrhage is difficult and its prognosis is especially poor. History of abdominal or pelvic surgery[1], or failure of cholangiojejunostomy or inappropriate reconstruction of the portal vein has been suggested as a cause of extrahepatic portal obstruction after reconstruction of the biliary tract. In our patient, reconstruction of the biliary tract was performed in the initial operation for cancer and there was no postoperative anastomotic leakage. Changes in transaminases were extremely small immediately after surgery, making it unlikely that portal vein obstruction occurred in the early postoperative period. Given the large reproductive hypertrophy of the remaining liver, we believe that the cause of extrahepatic portal obstruction was an excursion of the portal vein associated with increased liver capacity.

Dissection of the hepaticojejunostomy in the second surgery performed for rupture of jejunal varices disconnected communication of jejunal varices located close to the site of hepaticojejunostomy with the intrahepatic portal branch. At that time, this was considered to be the only portal flow to the liver, and therefore we prepared for postoperative hepatic failure. However, improvement was obtained by performing only serial adsorptions of bilirubin without the need for plasma exchange, even though hepatic failure occurred. This may have been because the portal route via the jejunal varices was not the only portal route into the liver, and the portal vein route via the left gastric vein may already have been present. We performed blocking of the jejunal route to the liver as a desperate remedy without confirming the existence of the portal vein route via the left gastric vein, since we had no guarantee that the patient would survive the surgery and have a good prognosis. However, we now believe that the patient recovered from hepatic failure because a new route developed from the jejunal vein to the intrahepatic portal branch. However, development of varices formed in the jejunal vein 5 years later.

In the third operation, we decided to perform splenectomy with the aim of decreasing the blood supply from the jejunal vein by decreasing the supply from the left gastric vein. As a result, the blood flow via the left gastric vein would be increased and that via the jejunal vein would be decreased at completion of surgery. We believed that the blood flow in the splenic vein was high, since the spleen was greatly expanded, and that these symptoms could be improved with decreased jejunal vein flow and an increase or maintenance of blood flow from the left gastric vein. Interventional blocking of the portal vein via jejunal varices might have been an alternative option, and this procedure has been reported[4]. If splenectomy only has an insufficient effect, blocking the route to the liver from the jejunal vein might be a possibility. However, there was a risk of hepatic failure because of insufficient intrahepatic portal flow due to inflow of a sclerotherapeutic agent, since there was communication with the intrahepatic portal branch via jejunal varices. The patient may not have survived such interventional therapy without blood flow from the jejunal vein and only with the route from the left gastric vein. If jejunal variceal rupture redevelops and results in large gastrointestinal bleeding, we will consider administration of a thrombotic substance to the jejunal varices and expansion of the blood flow route into the liver from the left gastric vein.

Jejunal varices in Roux limb after biliary anastomosis are rare; only 7 cases have been reported [2-7] in the English literature which is summarized in Table 2. There was another case[8] with jejunal varices in Roux limb, but this case had no biliary anastomosis. Three of them were bleeding from anastomotic site. In the first case[5], balloon dilation of the portal trunk and the superior mesenteric vein was possible. In the second case[4], varices of the anastomotic site was coiled, but blood still flowed into the intrahepatic portal vein, which suggested preservation of hepatoportal flow via the collaterals through the jejunal Roux-en-Y limb. In the last case[7], jejunal varicous vein was ligated without a well-thought-out plan. No case has any complication as they might have sufficient another portal flow. However, our case had hepatic failure due to insufficient portal blood supply after resection with reanastomosis. We think that easy blocking of varices is dangerous in case with anastomotic varices, and it is important to confirm and preserve collateral inflows to the liver.

**Table 2:** Prior series of varices after Roux-en-Y operation for biliary tree

**Author**	**Age/sex**	**Previous diagnosis**	**Previous surgery**	**Location of varices**	**Treatment**
**Johnson (1997) **[5]	39/M	Chronic pancreatitis	Cystoduodenostomy, choledocho-jejunostomy	Hepato-jejunostomy	Stenting (portal vein)
**Ishida (1998) **[6]	75/F	Gallbladder cancer	Hepatojejunostomy	Jejunal loop	Resection of the jejunum
**Hiraoka (2001) **[2]	66/F	Pancreatic cancer	Pancreato-duodenectomy	Jejunal loop	Stenting (portal vein)
	44/F	Choledocho-lithiasis	Choledocho-jejunostomy	Jejunal loop	Shunting operation (jejunal vein to vena cava)
**Sato (2003) **[4]	79/F	Gallbladder cancer	Partial liver resection, cholecystectomy, bile duct resection, and hepaticojejunostomy	Jejunal loop (first), Hepato-jejunostomy (second:2 years later)	Resection of the jejunum (first), Coiling (jejunal vein) (second)
**Ota (2005) **[3]	64/M	Cancer of the papilla Vater	Pylorus preserving pancreato-duodenectomy	Jejunal loop	Stenting (portal vein)
**Smith (2006) **[7]	42/F	Chronic pancreatitis	Hepatojejunostomy, gastrojejunostomy, cholecystectomy	Hepato-jejunostomy	Ligation of dilated jejunal vein, repair of hepatojejunostomy
**Present case**	64/F	Hepatic hilar bile duct cancer	Right lobectomy of the liver, bile duct resection, and hepatojejunostomy	Hepato-jejunostomy (first, second)	Reconstruction of hepato-jejunostomy (first), Splenectomy (second)

Regardless, we believe that the case is already interesting in that we experienced two jejunal variceal ruptures at five-year intervals after extrahepatic portal obstruction.

## Competing interests

The authors declare that they have no competing interests.

## Authors' contributions

HT carried out all operations, the design of the study and drafted the manuscript, MM participated in intraoperative portography part, HA participated in the second operation part, NF participated in the second and third operation part, YM and TK were involved the clinical preparation of the patient. All authors read and approved the final manuscript.

## Consent

We have obtained written, informed patient consent for publication of the report and any accompanying images.
